# The combination of macleaya extract and glucose oxidase improves the growth performance, antioxidant capacity, immune function and cecal microbiota of piglets

**DOI:** 10.3389/fvets.2023.1173494

**Published:** 2023-07-27

**Authors:** Xing Chen, Fan Zhang, Huirong Li, Jie Liu, Yanping Jiang, Furong Ren, Libo Huang, Xuejun Yuan, Yang Li, Weiren Yang, Chongwu Yang, Shuang Li, Ning Jiao, Shuzhen Jiang

**Affiliations:** ^1^College of Animal Science and Veterinary Medicine, Shandong Agricultural University, Taian, Shandong, China; ^2^Shandong Livestock Product Quality and Safety Center, Shandong, China; ^3^Zhongcheng Feed Technology Co., Ltd., Feicheng, Shandong, China; ^4^Ciyao Animal Husbandry Station, Ningyang, Shandong, China; ^5^Guelph Research and Development Center, Agriculture and Agri-Food Canada (AAFC), Guelph, ON, Canada; ^6^Challenge Biotechnology Co., Ltd., Beijing, China

**Keywords:** macleaya extract, glucose oxidase, growth performance, cecal microbiota, piglets

## Abstract

This study aims to investigate the effects of macleaya extract and glucose oxidase combination (MGO) on growth performance, antioxidant capacity, immune function, and cecal microbiota in piglets. A total of 120 healthy 28-day-old weaned piglets were randomly divided into two treatments of six replicates. Piglets were either received a basal diet or a basal diet supplemented with 250 mg/kg MGO (2 g/kg sanguinarine, 1 g/kg chelerythrine, and 1 × 10^6^ U/kg glucose oxidase). The results showed that MGO supplementation increased average daily gain (ADG) and decreased feed:gain ratio (F/G) (*p* < 0.05). MGO increased serum superoxide dismutase (SOD) and glutathione peroxidase (GSH-Px) activity, and immunoglobulin G (IgG) content (*p* < 0.05), but decreased malondialdehyde (MDA) and interleukin 1β (IL-1β) content (*p* < 0.05). The jejunal mRNA expression of *nuclear factor erythroid 2-related factor 2* (*Nrf2*), *glutathione peroxidase 1* (*GPX1*), and *heme oxygenase 1* (*HO-1*) were increased in MGO group (*p* < 0.05), while that of *kelch like ECH associated protein 1* (*Keap1*) was decreased (*p* < 0.05). The Firmicutes was significantly increased at phylum levels in MGO group (*p* < 0.05). In conclusion, 250 mg/kg MGO improved piglet growth, and regulated intestinal flora of piglets, which provided a theoretical basis for MGO as an alternative additive for antibiotics.

## Introduction

Antibiotics have been used as feed additives in piglets to enhance their growth performance, promote intestinal health and improve immunity (1). However, with the ban on the use of antibiotics in feed, piglets are now faced with oxidative stress and inflammation, leading to growth restriction, disease, and even death (2). Hence, there is an urgent need to explore a alternative antibiotic substitutes than can improve the antioxidant capacity and immune function in piglets (3).

The macleaya extract (MCE) contains sanguinarine and chelerythrine, which possess anti-inflammatory, antioxidant, bactericidal, and anti-tumor properties (4). Accordingly, MCE plays roles in improving animal immunity and animal growth, and preserving intestinal health (5). Studies have shown that macleaya extract can improve the growth performance, antioxidant capacity, and immune function of piglets (6, 7) and broiler chickens (8). Previous study showed that 50 mg/kg macleaya extract containing 1.5% sanguinarine increased average daily gain (ADG) of piglets (7). Moreover, supplement 50 mg/kg macleaya extract in piglet’s diet increased ADG and average daily feed intake (ADFI) (Ref). In addition, the content of cecal microbiota, such as *Escherichia coli* and *Salmonella*, were significantly reduced (9). Previous study reported that 40 mg/kg macleaya extract could increase the immunoglobulin G (IgG) content in the serum of piglets on the seventh day of growth (10). Dietary supplementation of MCE (50 mg/kg) also increased the superoxide dismutase (SOD) activity, and decreased the malondialdehyde (MDA) content in piglet serum (11).

Glucose oxidase (GOD) is an aerobic dehydrogenase that originates from the fermentation of specific fungal strains, including *Aspergillus* and *Penicillium* (12). This enzyme is capable of oxidizing β-D-glucose into gluconic acid, while also producing hydrogen peroxide (HP) through the consumption of oxygen (13). HP can inhibit the growth of harmful gut bacteria and prevent bacterial invasion (14). Therefore, the characteristics of GOD, such as acid production, deoxygenation and sterilization indicate its potential as a substitute additive for antibiotics (15–17). Previous study have shown that 100 U/kg GOD supplementation could improve the growth performance of piglets (18). In addition, dietary supplementation of 3,000 U/kg GOD can increase the total superoxide dismutase (T-SOD) and glutathione peroxidase (GSH-Px) activity in the jejunum of piglets (19). Research shown that 200 mg/kg glucose oxidase reduced the ETEC induced decrease of IgG content and the abundance of *Lactobacillaceae* and *Lactobacillus salivarius* in the intestine (13).

Therefore, MCE or GOD can improve piglet growth performance, antioxidant capacity, immune function and gut microbiota structure. However, the effects of the combination of MCE and GOD on piglet physiology have not been reported. The objective of this study was to investigate the impact of MCE, which includes sanguinarine and chelerythrine, combined with GOD on growth performance, antioxidant capacity, immune function, and cecal microbiota in piglets. The findings of this study could serve as a theoretical basis for the efficacy of MCE-GOD compound additives on piglets.

## Materials and methods

### Experimental design and layers management

Piglets used in this experiment were cared in accordance with the guidelines for the care and use of laboratory animals described by the Guide for the Care and Use of Laboratory Animals and approved by the Committee on the Ethics of Shandong Agricultural University (SDAUA-2020-0710).

A total of 120 healthy 28-day-old weaned piglets (Duroc × Large White × Landrace) were randomly divided into two treatments of six replicates, and with ten piglets per replicate. The control group was fed a basal diet, while the experimental group was fed a basal diet supplemented with 250 mg/kg of MGO (a combination of MCE and GOD). The MGO contained 2 g/kg of sanguinarine, 1 g/kg of chelerythrine, and 1 × 10^6^ U/kg of GOD. The MGO was provided by Shengdao Biological Co., Ltd. (Taian, Shandong, China). The feeding trial lasted for 35 days after 7-d adaptation at a piggery of Shandong Agricultural University. Piglets have free access to feed and water. The piglets were feed with powdered feed, and the treatment group’s feed was mixed with 250 mg/kg of MGO before feeding, ensuring an even distribution of the additive. The experimental diets were formulated according to National Research Council (20). The composition and calculated nutrient levels in the basal diet were shown in Table 1. Before feeding, diets were completed, sampled, and stored in covered containers. The piggery was thoroughly cleaned and disinfected before the experiment, and the pigs were disinfected once a week during the experimental period. During the first week, the ambient temperature was kept at about 30°C, and then maintained between 26°C to 28°C until the end of experiment. The relative humidity of the pig house was approximately 65%.

**Table 1:** Dietary components and nutrient levels in the basal diet (as fed-basis).

Ingredients	Content	Nutrient level	Content
Corn	67.75	Digestive energy, MJ/kg	14.63
Soybean meal 46% crude protein	10.00	Crude protein	18.89
Plasma protein	2.10	Calcium, %	0.70
Full-fat soybean	9.00	Phosphorus	0.60
Fermented soybean meal	6.80	Apparent total tract digestibility phosphorus	0.30
CaHPO_4_	1.09	Standardized total tract digestible phosphorus	0.42
Limestone	1.10	Lysine	1.41
NaCl	0.45	Methionine	0.46
Lysine	0.40	Methionine + Cysteine	0.67
Methionine	0.16	Threonine	0.88
Threonine	0.15	Thrptophan	0.22
Premix^a^	1.00		
Total	100		

a Premix provided the following per kilogram of diet: VA 2300 IU; VD_3_ 230 IU; VE 20 IU; VK_3_ 0.60 mg; VB_1_ 1.80 mg; VB_2_ 4.25 mg; Pantothenic acid 13.00 mg; Nicotinic acid 20.00 mg; Pyridoxine 2.00 mg; Biotin 0.09 mg; Folic acid 0.45 mg; VB_12_ 0.02 mg; Mn (Mn-Methionine) 6.00 mg; Fe (Iron(II) fumarate) 150 mg; Zn (Zn-Glycine) 150 mg; Cu (Cu-Glycine) 9.00 mg; I (Calcium iodate) 0.21 mg; Se (Selenium-rich yeast) 0.45 mg.

### Sampling collection

At the begining and end of the experiment, the body weight of each piglet was measured. The feeding intake of each replicate was recorded daily. The average daily gain (ADG), average daily feed intake (ADFI), and feed: gain ratio (F/G) were calculated.

At the end of the experiment, 10 mL blood was sampled from the jugular veins into tubes without anticoagulant after a 12-h fasting on the last day of the experiment. After centrifugation at 3,000 × *g* for 15 min at room temperature, the serum was obtained in 1.5 mL Eppendorf tubes.

One piglet with similar weight was selected from each repeat, and the head was electrocuted (110 V, 60 Hz) for 5 s to cause death. The 3 cm jejunum in the same part were isolated under sterile conditions from piglets randomly selected from each replicate. The removed jejunum was immediately frozen in liquid nitrogen and stored at −80°C for the subsequent analysis of mRNA expression. In addition, about 3 mL of cecal contents were stored at −80°C for microbial sequencing analysis.

### Determination of serum antioxidant, immunoglobulin and cytokines

Serum superoxide dismutase (SOD), glutathione peroxidase (GSH-Px) activity, and malondialdehyde (MDA) content were measured according to the methods of SOD assay kits (A001-3-2, WST-1 method), GSH-Px assay kits (A005-1-2, Colorimetric method), and MDA assay kits (A003-1-1, TBA method) (Jiangsu Nanjing Jiancheng Biotechnology Co., Ltd., Jiangsu, China). The measurement wavelengths of SOD, GSH-Px and MDA were 450 nm, 412 nm, and 532 nm, respectively. Serum immunoglobulin G (IgG, H106-1-1), immunoglobulin M (IgM, H109-1-2), immunoglobulin A (IgA, H108-1-2) and inflammatory factors including interleukin 1β (IL-1β, H002), interleukin 6 (IL-6, H007-1-2), interleukin 10 (IL-10, H009-1), and tumor necrosis factor-α (TNF-α, H052-1) were determined using commercial enzyme linked immunosorbent assay (ELISA) kits (Jiangsu Nanjing Jiancheng Biotechnology Co., Ltd., Jiangsu, China).

### Determination of relative mRNA expression in jejunum

The total RNA was extracted from the jejunum samples using RNAiso Plus (D9108B, Takara Bio Inc., Kusatsu, Japan) according to the manufacturer’s instructions. RNA purity and concentration were assessed using an Eppendorf Biophotometer (RS323C, Eppendorf Aktien Gesellschaft, Hamburg, Germany) with an absorbance ratio of 260/280 nm (range 1.8–2.0 represents pure RNA samples). The RNA integrity was verified by agarose gel electrophoresis. Total RNA was reverse transcribed to cDNA using the Reverse Transcription System Kits (Prime-Script RT Master Mix, RR036A, Takara Bio Inc., Kusatsu, Japan). The cDNA is used for polymerase chain reaction (PCR).

The total volume of the PCR mix was 20 μL, containing 10 μL SYBRY Premix Ex Taq II, 0.4 μL DyeII (SYBRY Premix Ex Taq-TIi RNaseH Plus, DRR420A, Takara Bio Inc., Kusatsu, Japan), 0.4 μL forward primers, 0.4 μL reverse primers, and 2 μL of cDNA (< 100 ng) for quantitative real-time PCR (qRT-PCR) analysis. The optimized qRT-PCR protocol consisted of an initial denaturation step at 95°C for 30 s, followed by 43 cycles of 95°C for 5 s, 60°C for 34 s, 95°C for 15 s, 60°C for 60 s, and 95°C for 15 s. The qRT-PCR was performed using the AB 7500 Real-Time PCR System (Applied Biosystems, Foster City, United States). The relative mRNA expression levels of *kelch like ECH associated protein 1* (*Keap1*), *nuclear factor erythroid 2-related factor 2* (*Nrf2*), *glutathione peroxidase 1* (*GPX1*), and *heme oxygenase 1* (*HO-1*) were calculated by the 2^-△△CT^ method, and each sample was analyzed three times in replicates. The primer sequence and product size are shown in Table 2.

**Table 2:** Primer sequences used for quantitative real-time PCR.

Genes	Primer sequence(5′–3′)	Product size bp	Location
*Keap1*	F: GTGTTACTACCCAGAGAGGAATGA	104	NM_001114671.1
	R: CCGCAGCATAGATACAGTTGTG		
*Nrf2*	F: CCAGTCTTCATTGCTCCTAACCA	109	XM_013984303.2
	R: CCTCCCAAACTTGCTCAATATCCT		
*HO-1*	F: CCGCCTTCCTGCTCAACATTC	80	NM_001004027.1
	R: CGAGGGTCTCTGGTCCTTAGTG		
*GPx1*	F: ACCTATGTGGAGGAACACCTGATG	96	NM_214201.1
	R: AGGAGCTGTGGTCTGGGAAAG		
*β-actin*	F: GGACTTCGAGCAGGAGATGG	138	XM_021086047.1
	R: AGGAAGGAGGGCTGGAAGAG		

*Keap1*, kelch like ECH associated protein 1; *Nrf2*, nuclear factor erythroid 2-related factor 2; *GPX1*, glutathione peroxidase 1; *HO-1*, heme oxygenase 1.

### Determination of cecal microbial sequencing

The total genomic DNA from the samples was extracted according to CTAB method. The DNA integrity and purity were determined using 1% agarose gel electrophoresis and a NanoDrop 2000 Spectrophotometer (Thermo Scientific, Waltham, United States). DNA concentration was accurately quantified by a Qubit Fluorometer (Thermo Scientific, Waltham, United States) and then diluted to 1 ng/μL with sterile water. The sequenced region of RCR amplification was V3-V4, and the primer sequences were 341F (CCTACGGGGRBGCASCAG) and 806R (GGACTACNNGGGTATCTAAT). The total volume of the PCR reaction system was 30 μL, consisting of Phusion^®^ High-Fidelity PCR Master Mix with GC Buffer 15 μL, Phusion^®^ High-Fidelity DNA Polymerase 0.5 μL (New England Biolabs, Herts Hitchin, UK), 1 μL upstream, 1 μL downstream, 2 μL of 10 ng/μL genomic DNA, and 10.5 μL of sterile ultrapure water. The amplification program was as follows: pre-denaturation at 98°C for 1 min, denaturation at 98°C for 10 s, annealing at 50°C for 30 s, extension at 72°C for 30 s for a total of 30 cycles, stable extension at 72°C for 5 min, and finally at 4°C was stored (PCR instrument: ABI GeneAmp^®^ Model 9,700, Applied Biosystems, Foster City, Unites States). PCR products were pooled and detected by 2% agarose gel electrophoresis; then quantified by the Quantus^™^ Fluor-ST Fluorometric Quantitation System (Promega, Madison, United States). The resulting PCR products were concentrated, mixed in equal amounts, and re-electrophoresed on a 2% agarose gel. The target product bands were recovered using the QIAquick gel extraction kit (Axygen, Santa Clara Valley, United States). The library was constructed with TruSeq DNA PCR-free DNA library kit (Illumina, San Diego, United States), and sequenced with HiSeq2500 PE250 after qubit and qPCR quality control. The constructed library was quantified by Qubit, and then NovaSeq6000 was used for on-machine sequencing. After strict filtering and quality control screening, the sequences were clustered into Operational Taxonomic Units (OTUs) with 97% identity and the number of OTUs was calculated. OTUs clustering and species classification analysis were performed on the valid data.

### Statistical analyzes

Data analysis was conducted using the general linear model (GLM) in SAS 9.4 (SAS Institute Inc., Cary, NC, United States), with t-test utilized to compare differences among treatments. Mean and standard error of the mean (SEM) were presented as results. The level of *p* < 0.05 was used to determine differences. GraphPad prism 9.0 (GraphPad Software, San Diego, United States) was used for image production. The petal diagrams for the OTUs data were generated using R software version 3.0.3 and the Venn Diagram package. The Chao1, ACE, Shannon, and Simpson indices were calculated using Qiime software (Version 1.7.0) (3). PyNAST software (Version 1.2) was utilized for rapid multiple sequence alignment with the Core Set data from the SILVA database (21). The phylogenetic tree was constructed using FastTree software and the sequence alignment results were compared using the approximate maximum likelihood algorithm (22).

## Results

### Growth performance

**Figure 1:**
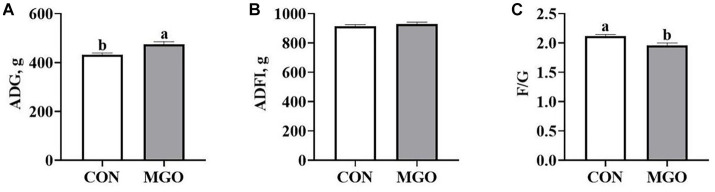
Effects of macleaya extract and glucose oxidase combination on growth performance of piglets. **(A–C)** Represent the average daily gain (ADG), average daily feed intake (ADFI), and feed:gain ratio (F/G), respectively. CON, basal diet; MGO, basal diet supplemented with 250 mg/kg macleaya extract and glucose oxidase (*n* = 6). Different lowercase letters in the figure indicate significant differences (*p* < 0.05).

The effect of MGO on growth performance of piglets is shown in Figure 1. Compared with the CON group, MGO group supplementation in the diet increased ADG (Figure 1A) (*p* < 0.05), and decreased the F/G (Figure 1C) (*p* < 0.05). However, no significantly were observed in the ADFI (Figure 1B) between the CON and MGO groups (*p* > 0.05).

### Serum antioxidant capacity

**Figure 2:**
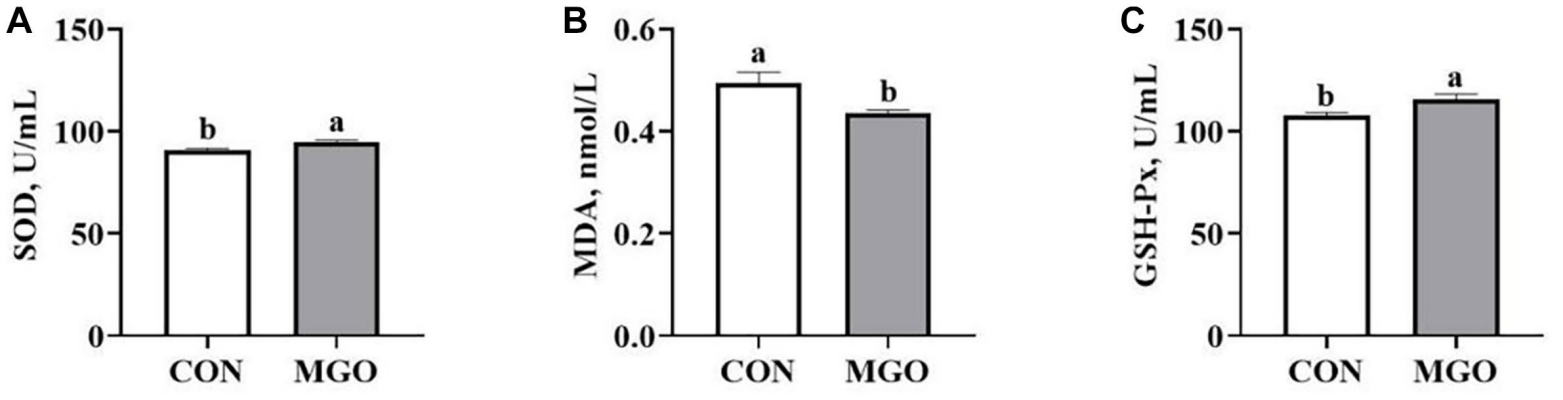
Effects of macleaya extract and glucose oxidase combination on serum antioxidant capacity of piglets. **(A–C)** Represent the serum superoxide dismutase (SOD), malondialdehyde (MDA), and glutathione peroxidase (GSH-Px), respectively. CON, basal diet; MGO, basal diet supplemented with 250 mg/kg macleaya extract and glucose oxidase (*n* = 6). Different lowercase letters in the figure indicate significant differences (*p* < 0.05).

The effect of MGO on the serum antioxidant capacity of piglets is shown in Figure 2. The increased serum SOD (Figure 2A) and GSH-Px (Figure 2C) activity (*p* < 0.05), and decreased MDA (Figure 2B) content in MGO group were observed compared with CON group (*p* < 0.05).

### Serum immunoglobulin content

**Figure 3:**
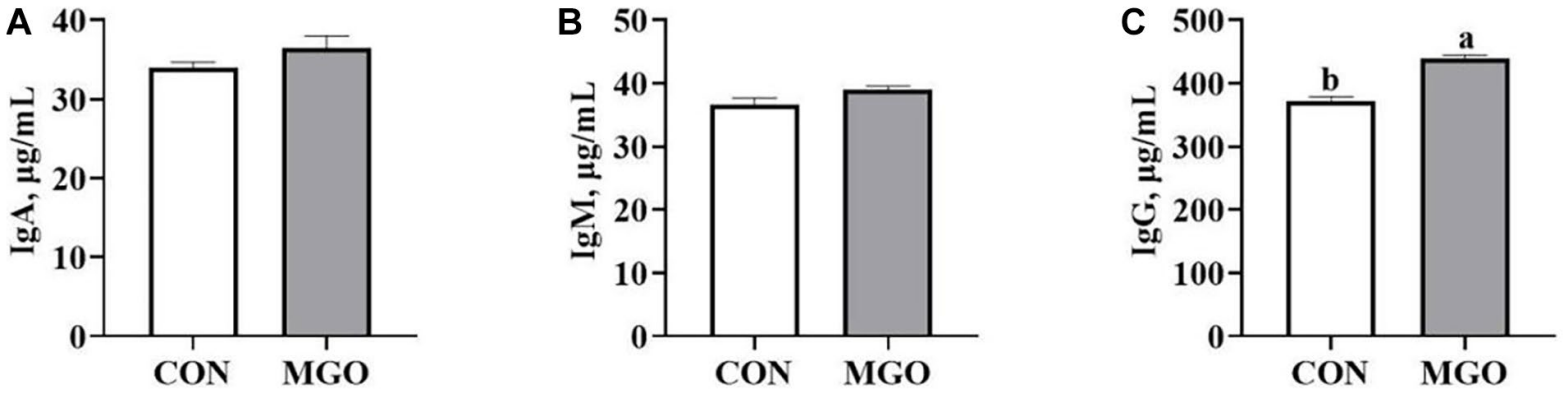
Effects of macleaya extract and glucose oxidase combination on serum immunoglobulin content of piglets. **(A–C)** Represent the serum immunoglobulin A (IgA), immunoglobulin M (IgM), and immunoglobulin G (IgG), respectively. CON, basal diet; MGO, basal diet supplemented with 250 mg/kg macleaya extract and glucose oxidase (*n* = 6). Different lowercase letters in the figure indicate significant differences (*p* < 0.05).

The effect of MGO on the serum immunoglobulin content of piglets is shown in Figure 3. The serum IgG (Figure 3C) content was significantly increased when MGO was added to the diet (*p* < 0.05). However, there were no significant differences in IgA (Figure 3A) or IgM (Figure 3B) content between CON and MGO groups (*p* > 0.05).

### Serum cytokine levels

**Figure 4:**
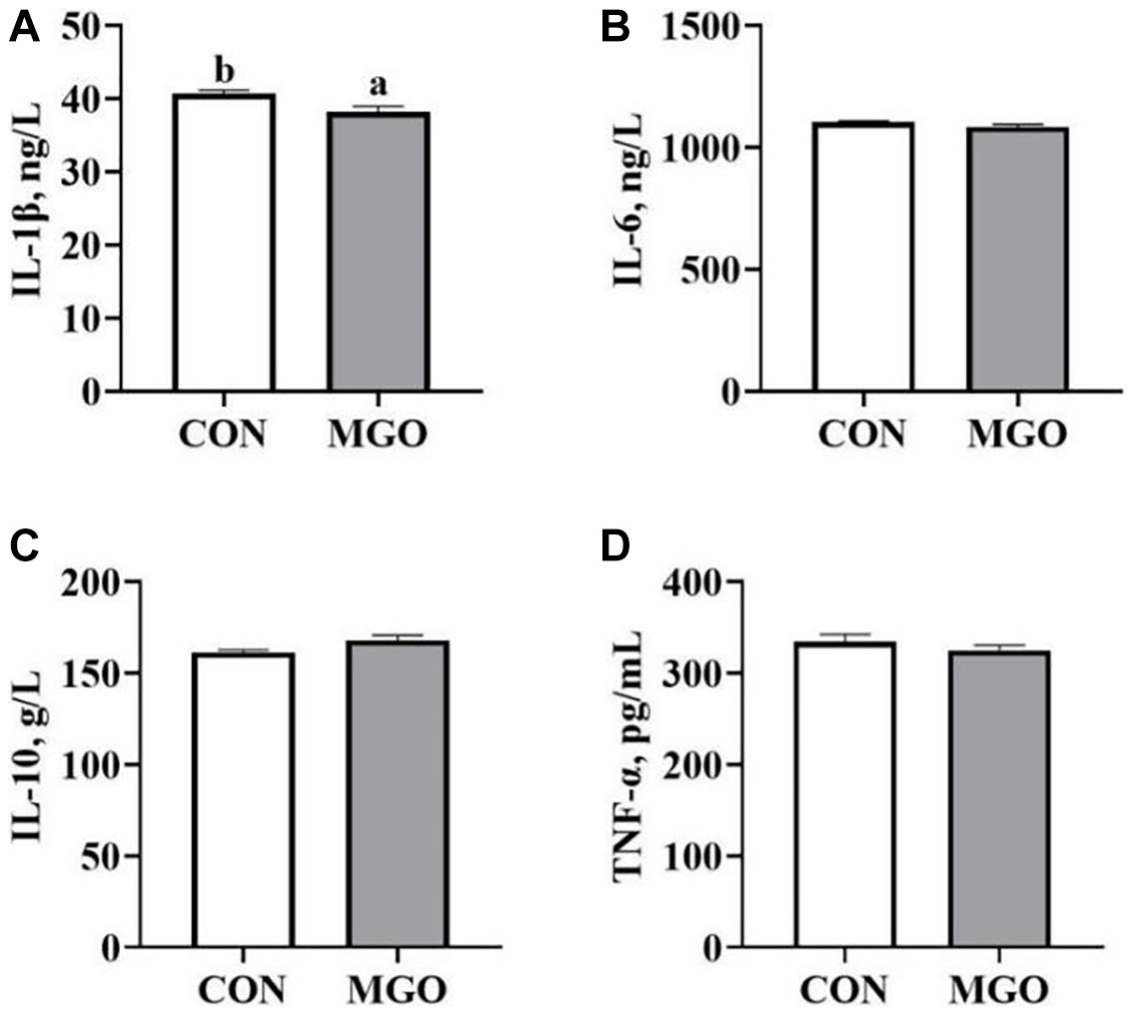
Effects of macleaya extract and glucose oxidase combination on serum cytokine levels of piglets. **(A–D)** Represent the serum cytokines Interleukin 1β (IL-1β), interleukin 6 (IL-6), interleukin 10 (IL-10), and tumor necrosis factor-α (TNF-α), respectively. CON, basal diet; MGO, basal diet supplemented with 250 mg/kg macleaya extract and glucose oxidase (*n* = 6). Different lowercase letters in the figure indicate significant differences (*p* < 0.05).

The Figure 4 showed the effect of MGO on the serum cytokine levels of piglets. Compared with the CON group, the serum IL-1β (Figure 4A) level was significantly decreased in the MGO group (*p* < 0.05), However, no significant changes were found in IL-6 (Figure 4B), IL-10 (Figure 4C) or TNF-α (Figure 4D) levels between CON and MGO groups (*p* > 0.05).

### Antioxidant mRNA expression

**Figure 5:**
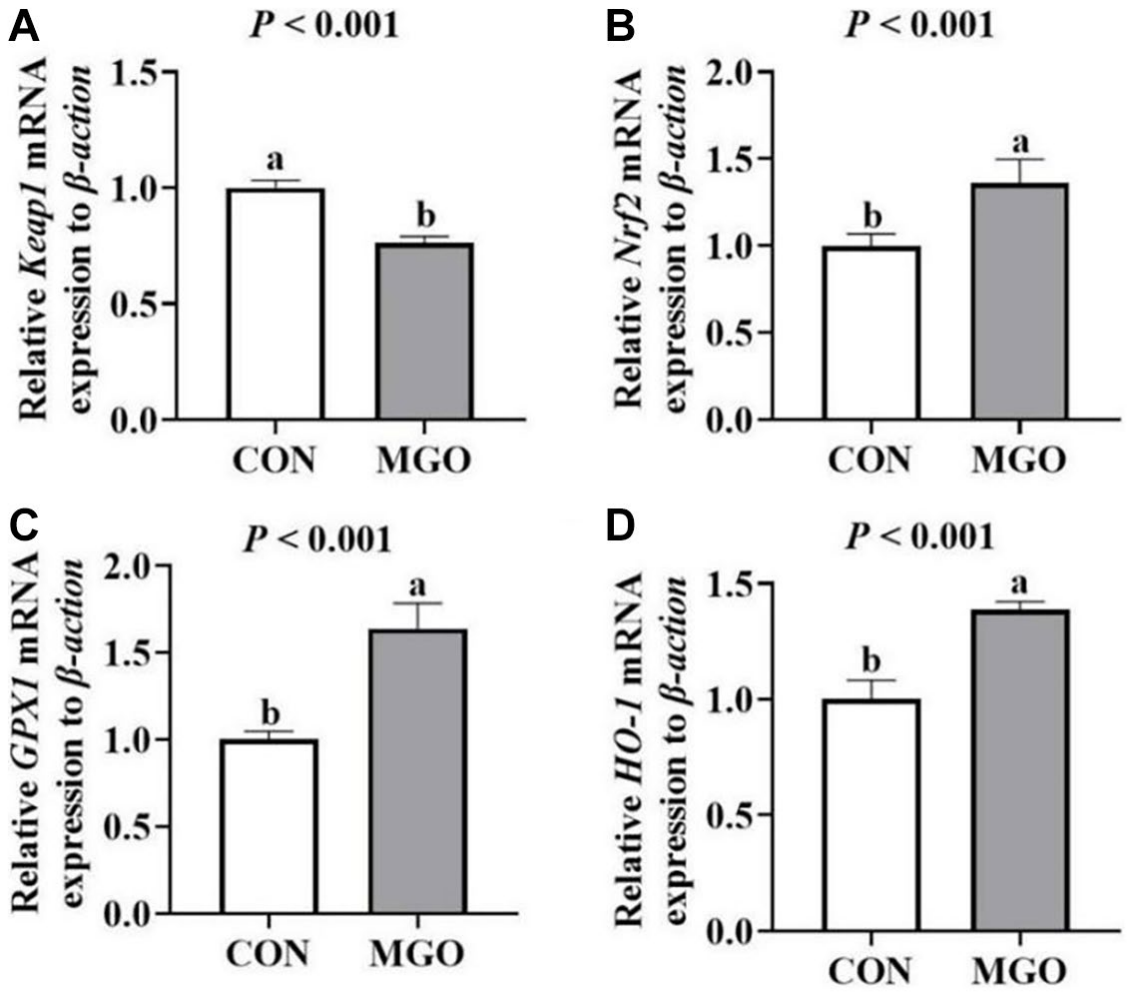
Effects of macleaya extract and glucose oxidase combination on the antioxidant mRNA expression of piglets. **(A–D)** Represent the jejunum antioxidant mRNA expression of *recombinant kelch like ECH associated protein 1* (*Keap1*), *nuclear factor erythroid 2-related factor 2* (*Nrf2*), *glutathione peroxidase 1* (*GPX1*), and *heme oxygenase 1* (*HO-1*), respectively. CON, basal diet; MGO, basal diet supplemented with 250 mg/kg macleaya extract and glucose oxidase (*n* = 6). Different lowercase letters in the figure indicate significant differences (*p* < 0.05).

As shown in Figure 5, supplementation of MGO in the diet significantly increased the mRNA expression of *Nrf2* (Figure 5B), *GPX1* (Figure 5C), and *HO-1* (Figure 5D) (*p* < 0.05). However, the mRNA expression of *Keap1* (Figure 5A) was decreased in MGO group compared to the CON group (*p* < 0.05).

### Microbial diversity analysis

#### Sequencing data analysis

**Figure 6:**
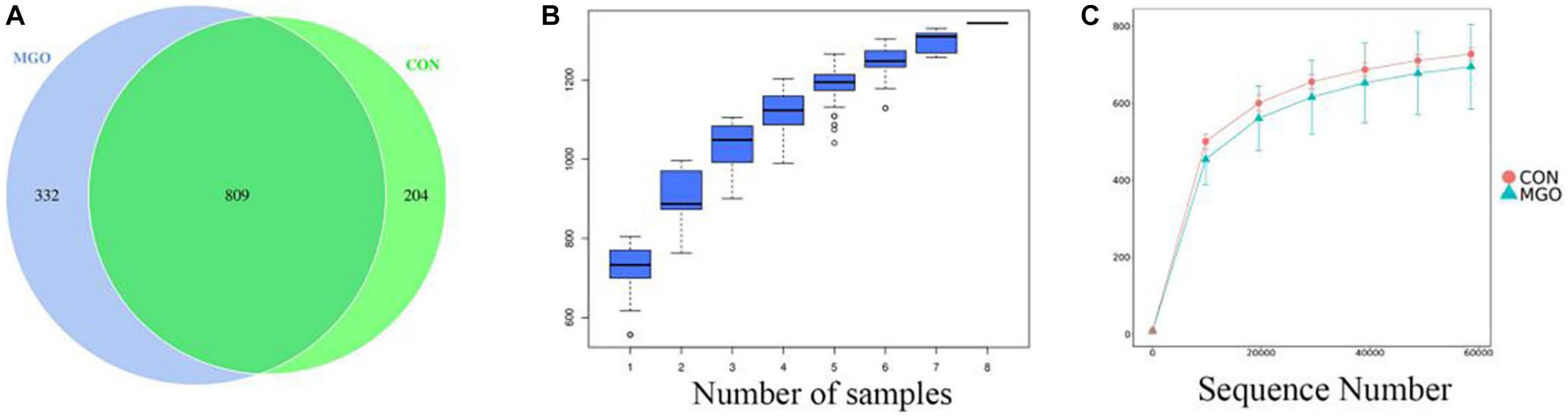
Differences in bacterial community diversity and richness. **(A–C)** Represent the cecal microbiota venn, species cumulative box, and species diversity curve by OTUs diagram drawn, respectively. CON, basal diet; MGO, basal diet supplemented with 250 mg/kg macleaya extract and glucose oxidase (*n* = 4).

There were 809 general OTUs (Figure 6A), among which the unique OTUs of CON and MGO groups were 204 and 332, respectively. The cumulative amount of sequencing data is shown as the species accumulation boxplot (Figure 6B) and rarefaction curve (Figure 6C). The results showed that the boxplots flatten out when the sample size reached 8. In addition, the curve tended to be flat when the number of randomly sampled sequences was close to 20,000, and the dilution curve tended to be saturated when the number of sequences was close to 60,000 for all samples. The curve lengths of the samples were similar, indicating that the number of sequenced samples sequenced was reasonable and could truly reflect the information of most microorganisms in the cecal contents.

#### Alpha diversity index analysis

**Figure 7:**
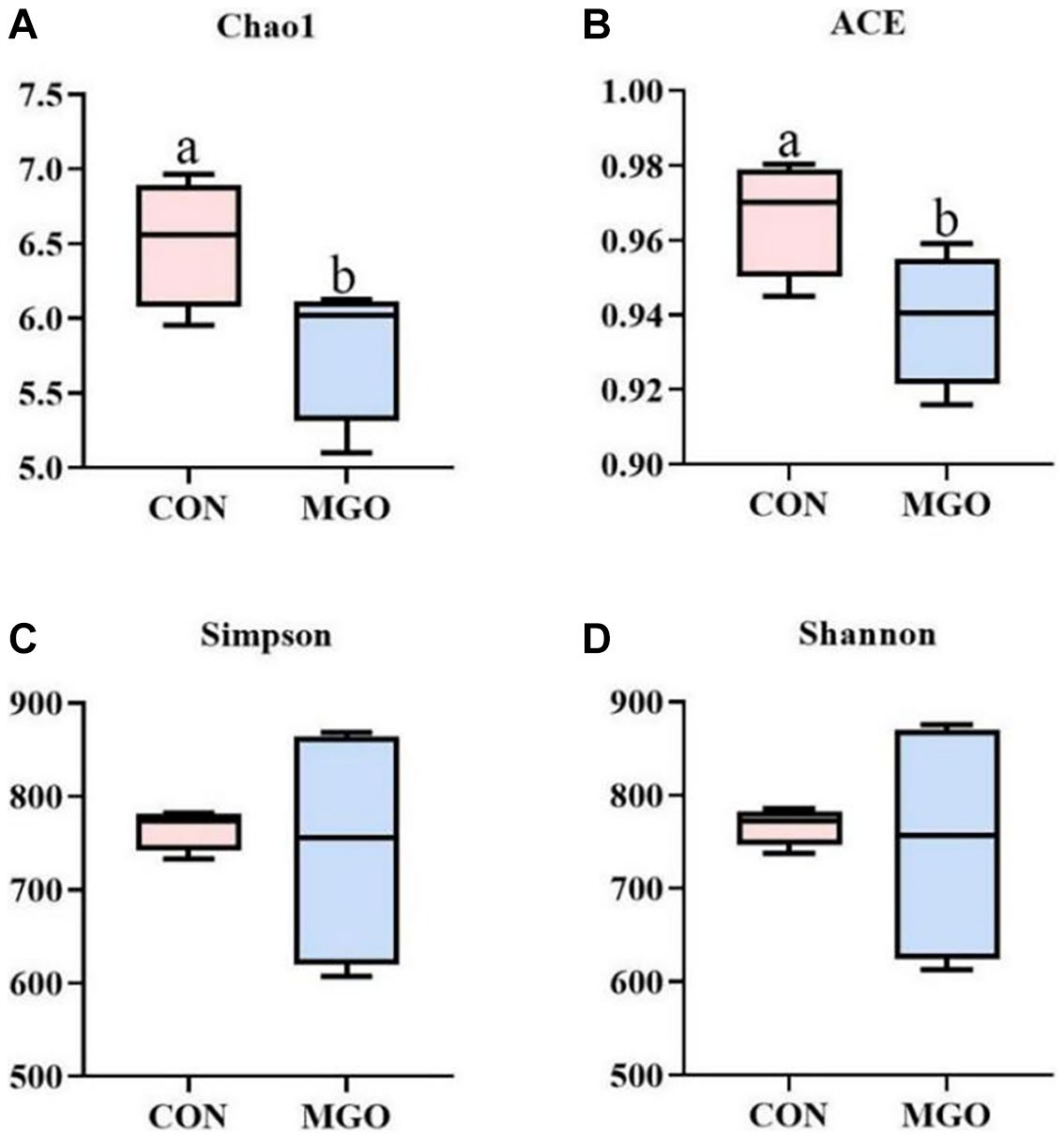
The alpha diversity index analysis. **(A–D)** Represent the cecal microbiota Chao1, ACE, Simpson, and Shannon index in alpha diversity index, respectively. CON, basal diet; MGO, basal diet supplemented with 250 mg/kg macleaya extract and glucose oxidase (*n* = 4).

In the alpha diversity index analysis (Figure 7), Chao1 (Figure 7A) or ACE (Figure 7B) indices represent bacterial abundance, and Simpson (Figure 7C) or Shannon (Figure 7D) indices represent bacterial diversity. In the present study, MGO supplementation significantly increased Chao1 and ACE indices (*p* < 0.05), but had no significant effect on Simpson and Shannon indices compared to the CON group (*p* > 0.05).

#### PCA and PCoA analysis

**Figure 8:**
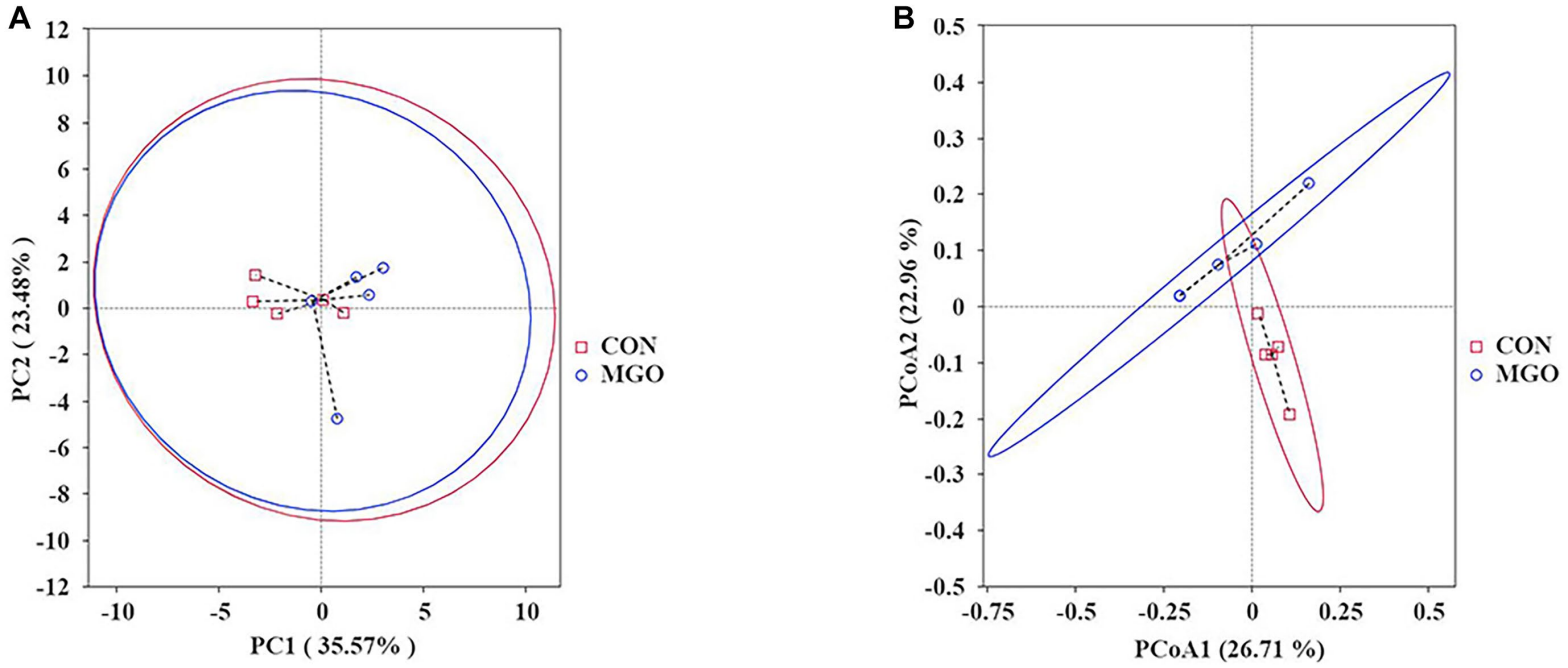
The beta-diversity index analysis. **(A,B)** Represent the cecal microbiota principal component analysis (PCA) and principal component coordinate analysis (PCOA) in beta-diversity index, respectively. CON, basal diet; MGO, basal diet supplemented with 250 mg/kg macleaya extract and glucose oxidase (*n* = 4).

In beta-diversity index analysis (Figure 8), the distance between samples on PCA (Figure 8A) and PCoA (Figure 8B) represents the degree of structural similarity in species composition. In the present study, PCA and PCoA indices were not significantly effect between CON and MGO groups (*p* > 0.05).

### Relative abundance of cecal microbiota

**Figure 9:**
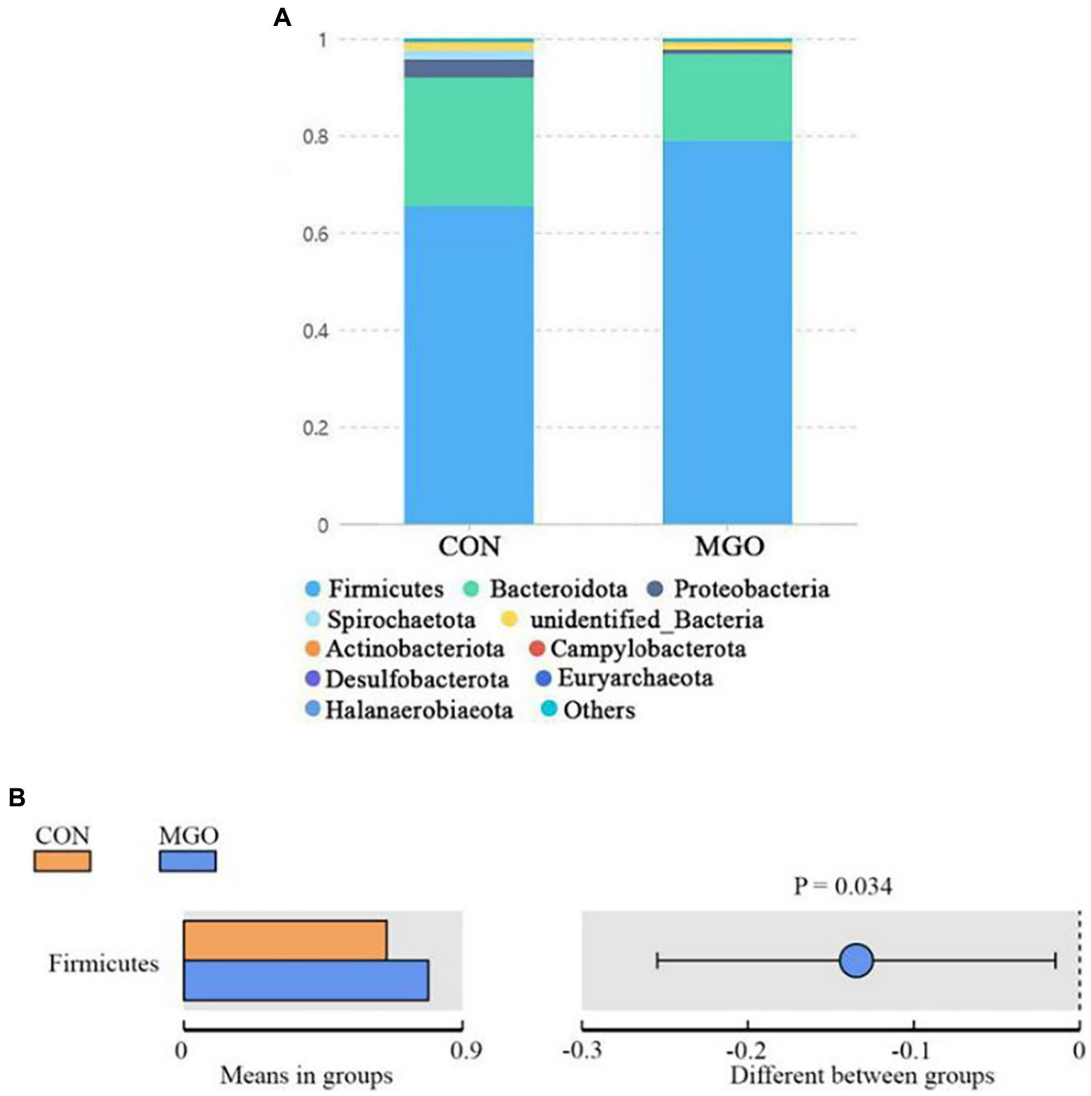
The relative abundance of species at phylum level. **(A,B)** Represent the phylum level relative abundance and T-test, respectively. CON, basal diet; MGO, basal diet supplemented with 250 mg/kg macleaya extract and glucose oxidase (*n* = 4).

The predominant phyla in cecal samples are Bacteroidetes and Firmicutes (Figure 9). The addition of MGO to the diet significantly increased the number of Firmicutes (Figure 9B) compared to the CON group (*p* < 0.05). However, the Bacteroidota, Proteobacteria, Spirochaetota, unidentified_Bacteria, Actinobacteriota, Campylobacterota, Desulfobacterota, Euryarchaeota, and Halanaerobiaeota (Figure 9A) were not significantly effect between CON and MGO groups (*p* > 0.05).

## Discussion

As the global prohibition of antibiotics, many researches paid attention to plant extracts compound additives as an alternative additive for antibiotics to improve animal growth performance. Previous study found that 120 mg/kg macleaya extract (a standardized premixture of MCE) increased weight gain and nutrient digestibility of piglets (23). Sureshkumar et al. reported that the ADG were significantly increased in piglets fed diet supplemented with 400 U/kg glucose oxidase (24). In the present study, MCE and GOD compound additives significantly increased ADG and decreased F/G, but did not change ADFI in piglets. The joint use of these supplements improved piglet growth and development, possibly due to their individual properties such as anti-inflammatory and antibacterial functions. In addition, MCE and GOD combination can improve feed utilization of piglets. However, the synergistic mechanism of the macleaya extract and glucose oxidase needs further confirmation.

It was remarkable that the activity of SOD and GSH-Px, and content of MDA content were the important markers of animal antioxidant capacity (3). MCE have been shown antioxidant effects because of their richness in sanguinarine and chelerythrine (25, 26). Wang et al. reported that 50 mg/kg MCE containing more than 3.75% sanguinarine could significantly increase serum SOD activity and decrease MDA content in piglets (11). GOD catalyzes glucose to produce gluconic acid and H_2_O_2_, which can inhibit harmful bacteria (15). Zhang et al. found that GOD (3,000 U/g) improved the T-SOD and GSH-Px activity in the jejunum of piglets (Zhang et al., 2020). We found that the serum SOD and GSH-Px acitvity of piglets were significantly increased in MGO group compared to the CON group, which was similar to previous studies. Moreover, the serum MDA content was significantly decreased. The Keap1-Nrf2 signaling pathway is vital in protecting cells from endogenous and exogenous stress (27, 28). External factors enhanced antioxidant capacity by activating the expression of related antioxidant gene (GPX1 and HO-1) mRNA expression of the Keap1-Nrf2 signaling pathway (29). GOD can significantly inhibit the expression of *Keap1*, and increase *Nrf2* expression (17). In addition, MCE induced Nrf2 accumulation in RAW264.7 cells of rat macrophages (30). It was worth noting that the mRNA expression of *Nrf2*, *Keap1*, *GPX1*, and *HO-1* in the jejunum of MGO group increased in accrodance with the activation of SOD and GSH-Px in the present study. The combination of MCE and GOD can improve the antioxidant capacity of piglets, while the mechanism behind this phenomenon still needs further research.

IgA, IgM, and IgG are considered as important markers of immune function, and the improvement of growth performance may be attributed to more balanced immune homeostasis (31). Li et al. found that supplementation of 500 mg/kg MCE containing 0.15% sanguinarine in the diet significantly increased the serum IgG and IgM contents of piglets (32). In addition, Liu et al. reported that dietary supplementation with MCE containing 0.15% sanguinarine significantly reduced the serum IL-1β and IL-6 contents of piglets (33). These results indicated that the immune effects of MCE may be partly attributed to the induction of immunoglobulin production and the repression of inflammatory responses. Previous study has shown that GOD affects immune function by blocking the expression of inflammatory factors and increasing the content of immunoglobulin to enhance humoral immunity (34). In the present study, the MGO group significantly increased the serum IgG level and decreased IL-1β content in piglets, which was consistent with previous studies. The combination of MCE and GOD can improve the immune function of piglets by reducing oxidative stress and inflammatory response.

Microbiota play important roles in in the regulation of intestinal nutrient metabolism, immunity and barrier function, which were considered as regulator of pig intestinal health and growth performance (22, 35). Chao1, ACE, Simpson, and Shannon indexes are important indicators of alpha diversity, which are used to evaluate microbiota community richness and diversity (36). In the present study, we found that MCE and GOD combination significantly decreased Chao1 and ACE index indicating the decrease of intestinal microbial community abundance, which may be caused by the antibacterial effect of MGO (37). Studies have shown that Bacteroides and Firmicutes are the major phyla in piglet cecum, which was consistent with the present study (38–40). In this study, it was found that piglet dietary supplementation of macleaya extract and glucose oxidase combination resulted in significant increase in the abundance of Firmicutes bacteria. Previous studies have shown that increasing the richness of Firmicutes in the cecum could increase the production of short-chain fatty acids, which can reduce the intestinal pH value and inhibit harmful bacteria growth (41–43). The combination of MCE and GOD can improve the composition of the cecal microbiota, thereby promote intestinal health in piglets.

## Conclusion

This study proposed that dietary MCE and GOD supplementation can enhance the growth performance, antioxidant capacity, and immune function of piglets. This can be attributed to an increase in the abundance of beneficial microbiota and a decrease in the abundance of harmful bacteria in the intestine. However, the synergistic molecular mechanism odf MCE and GOD in piglet growth, antioxidant capacity, and immune function need to be further confirmed by *in vivo* and *in vitro* studies.

## Data availability statement

The datasets presented in this study can be found in online repositories. The names of the repository/repositories and accession number(s) can be found at: https://www.ncbi.nlm.nih.gov/, PRJNA890335.

## Ethics statement

Piglets used in this experiment were cared in accordance with the guidelines for the care and use of laboratory animals described by the Guide for the Care and Use of Laboratory Animals and approved by the Committee on the Ethics of Shandong Agricultural University (SDAUA-2020-0710).

## Author contributions

XC and FZ: conceptualization and verification. FR, LH, XY, and NJ: data management. YJ and YL: formal analysis. WY and SJ: fund acquisition. YJ, CY, and SL: survey and resources. XC and NJ: methods. WY, NJ, and SJ: project management. FZ and YL: software. WY: supervision. NJ and SJ: visualization. XC: writing – manuscript. HL, JL, LH, XY, YL, CY, NJ, and SJ: writing – review and editing. All authors contributed to the article and approved the submitted version.

## Funding

This research was supported by Shandong Provincial Science and Technology SME Innovation Capacity Improvement Project (grant no: 2022TSGC1275) and Shandong Provincial Pig Industry Technology System (grant no: SDAIT-08-05).

## Conflict of interest

YJ was employed by Zhongcheng Feed Technology Co., Ltd. SL was employed by Challenge Biotechnology Co., Ltd.

The remaining authors declare that the research was conducted in the absence of any commercial or financial relationships that could be construed as a potential conflict of interest.

## Publisher’s note

All claims expressed in this article are solely those of the authors and do not necessarily represent those of their affiliated organizations, or those of the publisher, the editors and the reviewers. Any product that may be evaluated in this article, or claim that may be made by its manufacturer, is not guaranteed or endorsed by the publisher.
